# Assessing the Fortification Quality of Refined Vegetable Oil with Vitamin A, Wheat Flour with Iron, and Salt with Iodine: Findings from a Market Assessment in Senegal, West Africa

**DOI:** 10.1016/j.cdnut.2025.107440

**Published:** 2025-04-09

**Authors:** Mane Hélène Faye, Marie-Madeleine A Diémé, Phillip M Nkhoma, Adama Diouf, Dora Panagides, Abdou Badiane, Becky L Tsang, Olouwafemi Mistourath Mama, Marielle A De Souza, Nicole Idohou Dossou

**Affiliations:** 1 Laboratoire de Recherche en Nutrition et Alimentation Humaine, Département de Biologie Animale, Faculté des Sciences et Techniques, Université Cheikh Anta Diop, Dakar, Sénégal; 2 USAID Advancing Food Fortification Opportunities to Reinforce Diets, Arlington, VA, United States; 3 TechnoServe, Arlington, VA, United States; 4 Food Fortification Initiative, Atlanta, GA, United States

**Keywords:** compliance, fortification standards, wheat flour, oil, salt, iron, vitamin A, iodine, market assessment, Senegal

## Abstract

**Background:**

Mandatory fortification (MF) of wheat flour (WF) with iron/folic acid, refined edible oil with vitamin A (VA), and salt with iodine is implemented to address micronutrient deficiencies in Senegal. The effectiveness of fortification depends on food vehicles meeting fortification standards (FS).

**Objectives:**

The objective of this study is to assess iron, VA, and iodine content of WF, oil, and salt, respectively, and compare them with FS.

**Methods:**

A cross-sectional market survey was conducted in the Dakar region and the neighboring city of Thiès. Samples of all available brands of WF, oil, and salt in the scope of MF were collected at 25 sampling sites. Qualitative tests were conducted, and positive samples were pooled by brand and type, and then analyzed using atomic absorption spectrophotometry (iron), iCheck Chroma3 (VA), and titration (iodine). Micronutrient content of food vehicles was compared with FS.

**Results:**

Overall, 142 WF, 372 oil, and 140 salt samples were collected from 27, 69, and 31 brands, respectively. The qualitative results showed that 26.8% of WF, 44.6% of oil, and 23.6% of salt were not fortified. After quantitative analysis, 51.4% of WF, 17.3% of oil, and 16.3% of salt were found fortified below the standard minimum. The positive brand composite samples had the following median contents of iron, VA, and iodine: 17.5, 10.8, and 33.3 mg/kg, in WF, oil, and salt, respectively, and which were below, around, and above the minimum value of the corresponding standards, respectively. Disparities were observed by the origin of production, between domestic producers, and by type of food vehicle.

**Conclusions:**

Fortification quality gaps remain an issue in Senegal, and further action is needed to comply with FS and to realize the potential of food fortification for public health.

## Introduction

Micronutrient deficiencies are widespread worldwide, and young children and women of reproductive age are the most at-risk groups [1,2]. Latest estimates suggest that 56% and 69% (equivalent to 372 million preschool-aged children and 1.2 billion nonpregnant women of reproductive age, respectively) are deficient in ≥1 of 3 micronutrients [3]. Iron, iodine, folate, and vitamin A are among the micronutrients with the highest estimated prevalence of inadequate intake and their deficiencies are among those of major public health concern [[4], [5], [6], [7]]. These deficiencies contribute to poor growth, lower educational attainment, and impaired economic productivity, and are major causes of morbidity and, in extreme conditions, even mortality [8].

Food fortification is the practice of adding vitamins and minerals to foods to improve their nutritional quality and provide public health benefits with minimal risk to health. It is considered a cost-effective approach to increase the intake of micronutrients due to its potential to reach the majority of people in a population [6,9,10]. The efficacy of food fortification, as well as its effectiveness in improving micronutrient status, thus leading to a decline in the prevalence of corresponding deficiencies and functional health outcomes, is well established when programs are implemented with the appropriate conditions [[10], [11], [12]].

When large-scale food fortification (LSFF) is made mandatory, manufacturers are legally required to add micronutrients to widely consumed foods, such as staple foods, according to a specified target [9]. The success and public health impact of mandatory LSFF programs depend on high population coverage and compliance of fortified foods to standards. Compliance data are essential to inform fortification program improvements, such as identifying producers to target for improvements and understanding why gaps in micronutrient adequacy may still exist [13]. Using compliance data to monitor progress allows decision-making to ensure that impact is attained and sustained. Ideally, compliance data are collected through regulatory monitoring, which is defined as the continuous collection and review of information at key delivery points (production sites, customs warehouses, or market) to ensure fortified foods meet national nutrient fortification standards (FS) [14]. Unfortunately, external and commercial regulatory monitoring, which refers to monitoring by relevant government regulatory authorities, is often limited in countries due to various challenges, and even when implemented, inspection reports are not publicly available due to confidentiality reasons. Challenges to regulatory monitoring include inconsistent funding and capacity of regulatory agency (limited staff, e.g. inspectors and laboratory analysts, insufficient training, difficulties related to local availability/cost of equipment and reagents, etc.), as well as low priority for enforcement [[15], [16], [17]]. As a result, there are large gaps in compliance information. According to the Global Fortification Data Exchange, compliance data are only available for wheat flour (WF), oil, and salt in 10.9%, 5.7%, and 9.5% of countries with mandatory fortification programs, respectively [18]. Given the limited availability of compliance data, many nongovernmental entities assess the fortification quality of foods by collecting foods available in the marketplace (referred to as “quality” data, under a market assessment) [17]. Quality data are not formal compliance data that can be used to take enforcement action against the industry if FS are not met. However, it does provide additional information to identify where the gaps are. A few methodologies have been developed for this purpose [16,19]. Market assessments have been implemented in Burkina Faso, Bangladesh, Mozambique, Pakistan, Mexico, Nigeria, Afghanistan, and Uganda to fill compliance gaps [[20], [21], [22], [23], [24], [25], [26], [27], [28], [29]].

As part of its nutrition policy and strategy [[30], [31]], Senegal has a national fortification program that aims to reduce the most widespread micronutrient deficiencies by fortifying WF with iron and folic acid and refined edible oil with vitamin A [32]. These vehicles were identified on the basis of their extensive consumption in relatively high quantities in all Senegalese geographical and socioeconomic subgroups following a Fortification Rapid Assessment Tool survey [33]. An enabling environment for food fortification was established through: *1*) the creation of a national fortification alliance, *Comité Sénégalais pour la Fortification des Aliments en Micronutriments* (COSFAM), in 2006 to coordinate food fortification, *2*) the adoption of national FS for vitamin A in refined edible oil and for iron and folic acid in WF through the *Association Sénégalaise de Normalisation* (ASN) in 2008, *3*) mandating the use of these standards through legislation in 2009. These efforts complemented the country's commitment to combating iodine deficiency disorders, which had already resulted in the mandatory iodization of salt, endorsed by a ministerial order in 1995 and then followed by a decree in 2000 [32]. National standards were subsequently aligned with Economic Community of West African States (ECOWAS) standards [[34], [35], [36]]. However, Senegal still faces the challenge of micronutrient deficiencies among the most vulnerable population groups [37,38].

A 2013 Fortification Assessment Coverage Toolkit (FACT) survey revealed that household coverage for WF and oil, fortified at any level with iron plus folic acid and vitamin A, respectively, was only 51.2%, and 34.1%, respectively. This is despite the consumption of WF and oil in a fortifiable form by 81.5% and 95.0% of households [39]. Adequately iodized salt (≥15 ppm) only covered 37.2% of households (53.3% compared with 10.9% in urban and rural areas, respectively), whereas salt with any iodine was 81.3% [37]. Data on industry-level fortification compliance are only available for WF intended for use in bread production (96.0%) [40]. At the distribution level, the latest available market assessment dates back a decade [[41], [42]]. It indicates that 96.0% of WF and 76.1% of oil sold on the market were fortified to any level at the national level, but only 12.0% and 27.5% of these products met FS, respectively. An Industry Landscape Assessment in Senegal (through stakeholder interviews) estimated that refined oils are still likely to be inadequately fortified [USAID Advancing Food Fortification Opportunities to Reinforce Diets (USAID AFFORD) and Nicolas du Payrat (Altai Consulting), “Assessing Large-Scale Food Fortification Opportunities in Senegal: Industry feasibility of LSFF assessment,” 2023].

Regulatory monitoring to ensure compliance with fortified foods at all levels (import, production, and distribution) is the responsibility of the Directorate of Internal Trade, Ministry of Industry and Trade [43]. There are external and import monitoring protocols for oil and WF. COSFAM’s achievements under its first 2 strategic plans mention the quarterly implementation of regulatory monitoring by the above-mentioned entity, with the production of reports, including compliance rates for samples taken during inspections and quality control at distribution. However, details of this evidence are not publicly available [44]. In addition, consultations carried out as part of the third strategic plan preparation revealed persistent weaknesses in the quality control of fortified foods at the production level and an absence of control of the oil market at borders due to a lack of human resources for control throughout the country and insufficient training of control officers. The existing data landscape raises questions about the availability and quality of mandatorily fortified food vehicles. Despite investments made to enforce regulations and monitor the fortification process, it is unclear if the standards are being met. The objective of this market assessment of fortified foods was to assess, at the market level, the availability and amounts of iron, vitamin A, and iodine in fortified WF, refined edible oil, and salt, respectively, and how these align with the relevant FS.

## Methods

### Study design and setting

This study is designed as a cross-sectional market survey whose methodology was an adaptation of the Food Fortification Initiative’s PULL strategy in Uganda and Malawi and the Global Alliance for Improved Nutrition’s FACT methodologies while accounting for Senegal’s food distribution and marketplace context [16,19]. The methodology assumes that all targeted food vehicles and brands available elsewhere in Senegal are also readily available in Dakar [USAID AFFORD and Nicolas du Payrat (Altai Consulting), “Assessing Large-Scale Food Fortification Opportunities in Senegal: Industry feasibility of LSFF assessment,” 2023]. Therefore, the Dakar region and the neighboring city of Thiès were targeted and the survey was conducted from 10 to 14 August, 2023. Dakar is the main food processing and distribution center and port in Senegal, where wheat mills and refined oil industries are located and where 90% of foreign commercial imports are concentrated. The Thiès region is Senegal's second most populous region after Dakar, accounting for 35.7% of Senegal's population according to the latest census [45]. Its inclusion was assumed to maximize the number of fortified product brands potentially available on the market and accessible to a significant proportion of the population.

### Targeted food vehicles

This market assessment included food vehicles for which FS are mandatory by law in Senegal, in accordance with Decrees no. 2009-872 of 10 September, 2009 (WF and oil) and no. 2000/1154 of 29 December, 2000 (salt) (46,47]. Table 1 specifies the food vehicles covered by the standards and regulations.

**TABLE 1:** Senegal food fortification standards for selected nutrients and target fortification ranges.^1^^,^^2^^,^^3^

Food (reference of the standard)	Scope	Nutrient	Target range (mg/kg)^4^
Wheat flour (NS ECOSTAND-047: 2015)	Wheat flour intended for human consumption and derived from common wheat, *Triticum aestivum* L., or branched wheat, *Triticum compactum* Host. or any mixtures of these, which are prepackaged and ready for sale to consumers or for use in other food products.	Folic acid	2.3–2.9
		Iron (ferrous fumarate or sulfate)	54–66
		Iron (NaFeEDTA)^5^	36–44
Edible oil (NS ECOSTAND-008: 2014)	Cotton, palm kernel, palm, peanut, sesame, sunflower, rapeseed, corn and soybean oils.	Vitamin A	11–24
Salt (NS ECOSTAND-048: 2015)	Salt that is used as a food ingredient for both direct sales to the consumer and the food industry. It also applies to salt used as a carrier for food additives and/or nutrients (fortified).	Iodine (as potassium iodide or iodate)	20–60

1 For wheat flour, the range is for added iron and applies to all the wheat flour targeted by the standard, regardless of point of control.

2 For edible oil, the range refers specifically to the levels of vitamin A expected at the market level. The expected range at production and point of import is 16–24 mg/kg.

3 For salt, the range refers specifically to the levels of iodine expected at the market level.

4 mg/kg, milligrams of nutrient per kilogram of food.

5 NaFeEDTA, sodium ferric EDTA.

### Ethics

The study was approved by the Comité National d’Éthique pour la Recherche en Santé of the Senegalese Ministry of Health (reference: SEN23/66).

### Sampling and data collection

#### Sampling sites

A purposive sampling approach was used to identify 25 major sampling sites in Dakar and the city of Thiès from 5 marketplace categories: *1*) hypermarkets, *2*) retail outlets, *3*) open markets, *4*) wholesale outlets, and *5*) bakeries, with 5 sites visited per category. The selection of these sites, particularly hypermarkets and open markets, prioritized sites with a wider and more diverse range of food products, taking into account the different suppliers used, as well as the varying customer profiles and needs.

#### Food sampling and data collection

Food sampling and data collection were conducted by trained enumerators using an online form (KoboCollect), pilot-tested beforehand, and deployed on tablets. All food vehicle types included in the standards were eligible for sampling, whether locally produced or imported, and whether the products were branded or unbranded, packaged or loose/bulk.

Two samples of each brand and type of the 3 food vehicles of interest were collected in each supermarket, retail outlet, and bakery, when available. The smallest available package size for each packaged product was purchased, with a minimum content of 250 mL for oil and 300 g for WF and salt. In open markets, samples were collected in all outlets until no new brands were identified. At wholesale outlets, the smallest pack size was collected for each brand and type of food vehicle, and equal portions of the samples in the pack were later mixed to obtain the appropriate composite size sample. Samples of unbranded food vehicles were also collected in the same quantities.

Bulk samples were placed in clean, dry packaging. Oil samples were wrapped in aluminum foil for opacity. All samples were sealed to prevent contamination and leakage during transportation to the laboratory.

The initial stage of data collection enabled the gathering of basic information for each sample, such as the date, team number, name, and marketplace category, and details of the food vehicle, including its type, brand name, and code, as well as the sample number. This step facilitated the automatic generation of a unique sample identifier, which was written on a sticker and affixed to the sample on collection from the sampling site. The samples were transported to the laboratory in black plastic bags inside sealed boxes, to protect them from light and prevent spillage. The containers were labeled with the name and address of the sampling site. Additional information was extracted for each sample to complete the database. Additional information included: size (weight or volume) of the food vehicle container, type of packaging, fortification label (mention only, logo only, mention and logo, or no label), producer and/or supplier (importer, distributor, packer), place and date of production, batch number, and production and expiry dates. The samples were then stored at room temperature in the laboratory until analysis.

### Analyses of oil, wheat flour, and salt samples

#### Qualitative analysis of food samples

All food samples were first qualitatively analyzed for the presence or absence of added nutrients in the laboratory.

The oil was analyzed for the presence of vitamin A, specifically esters of retinol, using the method outlined in the East, Central and Southern Africa Health Community (ECSA-HC) manual [48]. The presence of iron, whether ferric or ferrous, in WF was determined using the iron spot test method described in the American Association of Cereal Chemists Method 40–40, as detailed in the USAID MOST Manual [49]. The Iodized Salt Test Kit for potassium iodate in iodized salt was used to test for iodine in the salt samples. If the iodate test was negative, then the iodide test was performed [50].

#### Preparation of composite samples

For each food vehicle, positive samples from the qualitative analysis were pooled by brand name and type to prepare composite samples. Samples were combined in equal amounts to create a composite sample of 250 g or 250 mL when there were 5 or fewer samples per brand. When the number of samples per brand exceeded 5, the preparation of additional composite samples of the same brand continued with the remaining samples, using the same approach. Bulk samples were composited as follows: *1*) samples from a known brand and producer were mixed with packaged samples of the same brand and type from the same producer; *2*) samples from an unidentified brand but known producer were mixed with bulk samples of the same type from the same producer; *3*) samples from an unidentified brand and producer were mixed with similar bulk samples from the same category of sampling sites.

#### Quantitative analysis of food samples

Quantitative analysis of food vehicles was conducted at the *Laboratoire de Recherche en Nutrition et Alimentation Humaine* (LARNAH), Dakar, Sénégal. Retinol concentration in refined oil was measured with the iCheck Chroma 3 device. The analytic method uses a colorimetric Carr–Price reaction combined with compensation for matrix effect. The sample reacts with a saturated solution made of chloroform and antimony trichloride. The linear range of the device is between 5 and 20 mg RE/kg [51]. BioAnalyt provided remote training, which was a refresher training, considering the LARNAH team’s previous experience using iCheck Chroma 3. The iron content in WF was determined by flame atomic absorption spectrophotometry after microwave pressure digestion [52]. For salt, the iodine content was determined by titration using the reference method [50]. Quality control was performed by measuring samples of oil, WF, and salt with known concentrations of vitamin A, iron, and iodine. A subset of samples was sent to an external reference laboratory (Medallion) for quality control before starting measurements at LARNAH.

#### Definitions of fortification quality categories

The concentrations of vitamin A in refined edible oil, total iron in WF, and iodine in salt composite samples for each brand were compared with the national standards defined by the ASN [[34], [35], [36]]. The required levels are presented in Table 1. For the purpose of this paper, iron content is used as a proxy indicator to assess the fortification status of folic acid in WF as folic acid was not measured for all the samples. On the basis of these thresholds, we established categories of fortification status: not fortified, fortified below the standard minimum, fortified within the standard range, and fortified above the standard maximum (Table 2).

**TABLE 2:** Fortification status categories.

Nutrient in food vehicle (unit)	Not fortified	Fortified		
		Below the standard minimum	Within the standard range	Above the standard maximum
Vitamin A in oil (mg retinol equivalent (RE)/kg)	Negative to the qualitative test	<11	11 ≤ Vitamin A ≤24	>24
Iron in wheat flour (mg/kg) 1	Negative to the qualitative test	<54	54 ≤ Iron ≤66	>66
Iodine in salt (mg/kg)	Negative to the qualitative test	<20	20 ≤ Iodine ≤60	>60

1 Assuming that ferrous sulfate or fumarate is used for fortifying wheat flour samples.

### Statistics

Data entry, processing, and analysis were carried out using KoboCollect (koboCollect version 2023.1.2), Microsoft Excel, and STATA/SE (version 14, Special Edition, Stata Corporation), respectively. The characteristics of the oil, salt, and WF samples found on the market, their respective concentrations of vitamin A, iodine, and iron, and their fortification quality according to Senegalese standards were determined using a descriptive analysis. For the purpose of this paper, the main producers of wheat flour, oil, and salt are anonymized as wheat flour producer (WFP), oil producer (OP), and salt producer (SP), respectively, and numbered (WFP1 to WFP6, OP1 to OP3, SP1, and SP2). However, it is important to note that in the case of wheat flour, WFP6 is not a miller, but a local producer of ready-to-use pastry mixes, and is therefore likely to be supplied by one of the local mills. The results are presented with and without his samples to better assess the compliance of the domestic mills involved in the LSFF. To estimate overall fortification quality, we assumed that each sample had the same content as the composite to which it had contributed, and we, therefore, applied the fortification status of composite samples to the individual samples from which they had been derived. Categorical variables were expressed as percentages and continuous variables were expressed as median with the IQR. The Wilcoxon rank-sum test or Kruskal–Wallis’s test was used to compare medians. Pearson's χ^2^ test or Fisher's exact test are used to compare percentages. Observations with missing outcome data were excluded from the analysis where needed. A *P* value <0.05 was used for significance for all statistical analyses.

## Results

### Characteristics of wheat flour, oil, and salt available in the marketplace

A total of 142 samples of WF from 27 different brands were collected (Table 3). A total of 77 samples (54.2%) were collected from hypermarkets. Pastry flour, bread flour, and all-purpose accounted for 26.1%, 21.1%, and 9.9% of samples, respectively. A total of 74.7% (*n* = 106) of the samples were domestically produced, whereas 23.2% (*n* = 33) were imported (with brands mainly from France and Germany). The name of the producer was listed for 88.7% (*n* = 126) of the flour samples. Samples from 5 major mills were found for domestic production. The majority of samples (78.9%) were collected in their original packaging. Samples were mainly packaged in plastic woven bags or sachets (50%) and paper bags (45.1%). The most common packaging sizes were 1 kg (38%), followed by samples purchased in bulk from 50 kg and 5 kg (20.4%) plastic woven bags, 800 g (18.3%), and 250 g (9.9%). Only 20.4% of the WF samples were labeled as iron-fortified, defined by the presence of a logo, a mention, or both. Of the samples labeled as fortified, 86.2% were bread flour.

**TABLE 3:** Characteristics of wheat flour samples.^1^^,^^2^

Characteristics	*n*	%
Marketplace categories		
Open markets	39	27.5
Hypermarkets	77	54.2
Retail outlets	13	9.2
Wholesale outlets	5	3.5
Bakeries	8	5.6
Type of wheat flour		
Bread	30	21.1
Pastry	37	26.1
All-purpose	14	9.9
Other types	61	43.0
Baking mixes	32	22.5
Type 45	7	4.9
Type 55	4	2.8
Type 65	14	9.9
Flowable	1	0.7
Not specified	3	2.1
Origin of production		
Domestically produced	106	74.7
WFP1	52	36.6
WFP2	8	5.6
WFP3	7	4.9
WFP4	6	4.2
WFP5	4	2.8
WFP6^3^	29	20.4
Imported	33	23.2
Unspecified	3	2.1
Packaging		
Original packaging	112	78.9
Loose form	30	21.1
Type of packaging		
Plastic sachet or woven bag	71	50.0
Paper bag	64	45.1
Cardboard box	7	4.9
Presence of fortification label		
No	113	79.6
Yes	29	20.4
Mention only	3	2.1
Logo only	24	16.9
Mention and logo	2	1.4

Abbreviation: WFP, wheat flour producer.

1 *N* = 142.

2 Values are *n* and %.

3 WFP6 is not a wheat flour mill but a manufacturer of pastry mixes for which the supplier mill is not identified. Only referred to WFP for the purpose of this paper.

Table 4 provides a comprehensive overview of the characteristics of the 372 oil samples that were from 69 brands. More than half (54.3%, *n* = 202) of oil samples were obtained from open markets. Only 4 of the 9 types of oil that are subject to mandatory fortification in Senegal were found in the marketplace, including sunflower oil (46.8%), soybean oil (27.2%), palm oil (19.9%), and peanut oil (3.8%). Only 24.7% (*n* = 92) of the samples were domestically produced. The majority of oil samples (90.9%) were purchased in their original packaging and were packed in clear plastic bottles (95.7%). The most common sizes were 250 mL (13.5%), 1 L (46.1%), and 5 L (27.8%). A fortification label was applied on the packaging of 97.9% (*n* = 364) of oil samples, including 70.9% (*n* = 258) with the fortification logo.

**TABLE 4:** Characteristics of oil samples.^1^^,^^2^

Characteristics	*n*	%
Marketplace categories		
Open markets	202	54.3
Hypermarkets	118	31.7
Retail outlets	31	8.3
Wholesale outlets	19	5.1
Bakeries	2	0.5
Type of oil		
Palm	74	19.9
Peanut	14	3.8
Sunflower	174	46.8
Soybean	101	27.2
Unspecified	9	2.4
Origin of production		
Domestically produced	92	24.7
OP3	12	3.2
OP1	42	11.3
OP2	32	8.6
Other domestic producers	6	1.6
Imported	262	70.4
Unspecified	18	4.8
Packaging		
Original packaging	338	90.9
Loose form	34	9.1
Type of packaging		
Clear plastic bottle	354	95.2
Dark plastic or glass bottle	10	2.7
Plastic sachet	6	1.6
Unspecified	2	0.5
Presence of fortification label		
Yes	364	97.9
Mention only	10	2.7
Logo only	258	69.4
Mention et logo	96	25.8
No	6	1.6
Unspecified	2	0.5

Abbreviation: OP, oil producer.

1 *N* = 372.

2 Values are *n* and %.

In the case of salt, 140 samples coming from 31 brands were found. A total of 86.4% of the samples were collected from open markets and hypermarkets. These samples were fine salt (67.1%) and coarse salt (30.0%), with 65.7% produced domestically. Almost 90% of the salt samples were collected in their original packaging (88.6%), with 86.5% of these samples packaged in plastic containers (woven bags, sachets, or pots). The packaging sizes were 250 g (27.1%), 500 g (17.1%), and 125 g (7.1%); the rest were served from 25 kg plastic woven bags. Overall, 87.1% of salt samples were marked with a fortification label, 98.9% for domestic samples, and 71.4% for imported samples, *P* < 0.001. Table 5 gives a detailed description of the characteristics of the salt samples. Characteristics of food vehicles in terms of brands are presented in Supplemental Tables 1 and 2.

**TABLE 5:** Characteristics of salt samples.^1^^,^^2^

Characteristics	*n*	%
Marketplace categories		
Open markets	63	45.0
Hypermarkets	58	41.4
Retail outlets	9	6.4
Wholesale outlets	5	3.6
Bakeries	5	3.6
Type of salt		
Fine salt	94	67.1
Coarse salt	42	30.0
Other	4	2.9
Origin of production		
Domestically produced	92	65.7
SP1	4	2.9
SP2	4	2.9
Other domestic producers	84	60.0
Imported	35	25.0
Unspecified	13	9.3
Packaging		
Original packaging	124	88.6
Loose form	16	11.4
Type of packaging		
Plastic sachet or woven bags	109	77.9
Plastic pot or bottle	12	8.6
Paper or cardboard container	18	12.9
Glass bottle	1	0.7
Presence of fortification label		
No	18	12.9
Yes	122	87.1
Mention only	82	58.6
Logo only	33	23.6
Mention and logo	7	5.0

Abbreviation: SP, salt producer.

1 *N* = 140.

2 Values are *n* and %.

### Fortification compliance of WF, oil, and salt available in the marketplace

The results of the qualitative test showed that overall, 73.2% (*n* = 104) of WF samples were fortified with iron, whereas 26.8% (*n* = 38) were not. Fortification compliance of wheat flour samples (70.8%, *n* = 80) did not differ with the exclusion of samples from WFP6 (*n* = 29). The proportion of iron-fortified samples was significantly higher among domestically produced WFs (83.1%) compared with imported ones (42.4%) (*P* <0.001). An analysis of locally produced samples showed that only WFP5 had all its samples fortified (*n* = 4). WFP1–WFP4 produced both fortified and unfortified flour but in different proportions. Over 90% and 87.5% of samples from WFP1 and WFP2, respectively, were fortified, compared with 42.9% and 50%, for WFP3 and WFP4, respectively. There was a significant difference in fortification compliance by WF type (86.7% for bread flour compared with 65.1% for nonbread flour, *P* <0.05). Overall, 82.8% of samples with a fortification label were found to be fortified with iron, which was not statistically different from samples without a fortification label (70.8%).

Regarding oil samples, 55.4% (*n* = 206) were fortified with vitamin A, whereas 44.6% (*n* = 166) were not. There was no statistical difference in the fortification compliance of domestic (48.9%) oils compared with imported (60.3%) oils. An analysis of the domestic samples revealed that all samples from OP3 (*n* = 12) and those of minor national producers (*n* = 6) were fortified with vitamin A, whereas only 37.5% (*n* = 12) and 35.7% (*n* = 15) of samples from OP2 and OP1, respectively, were fortified. In order of importance, fortification compliance was highest for peanut oil (100%, *n* = 14), palm oil (85.1%, *n* = 63), sunflower oil (58.6%, *n* = 102), and soybean oil (18.8%, *n* = 19). Fortification compliance of samples displaying a fortification label was 55.2%. All the samples packaged in dark plastic bottles were fortified with vitamin A (*n* = 10), whereas 46.3% of the samples packaged in transparent plastic bottles were not.

According to the qualitative test, 76.4% (*n* = 107) of salt samples were fortified with iodine, and 23.6% (*n* = 33) yielded negative results. The proportion of iodized salt samples produced locally was significantly higher than that of imported samples (85.9% compared with 62.9%, *P* <0.01). Among locally produced salt samples, all samples from major producers, SP1 (*n* = 4) and SP2 (*n* = 4), were iodized, compared with 84.5% of samples from small producers. A significantly higher proportion of salt samples bearing a fortification label were found to be fortified (84.4%) than those without (22.2%) (*P* <0.001).

The fortification compliance of WF, oil, and salt brands by origin of production and type of food vehicle is presented in Supplemental Table 3.

### Micronutrient content of food vehicles

Details of iron, vitamin A, and iodine content of WF, oil, and salt composite samples, respectively, by origin, type of food vehicle, and domestic producer are presented in Table 6. A total of 43 composite wheat flour samples from 23 brands, all of which had tested positive in the qualitative test, were prepared and assayed for iron by Atomic Absorption Spectrometry (AAS). Following analysis, 1 sample, which was identified as positive according to the qualitative analysis, was excluded as it presented an iron concentration suggesting intrinsic content (1 or 2 “spots” were seen in the qualitative test, which was still considered positive). The median iron content of composite samples was 17.5 mg/kg (14.3–26.8), whereas the Senegalese standard requires an iron content between 54 and 66 mg/kg when ferrous sulfate or fumarate is used, and 36–44 mg/kg if sodium ferric EDTA (NaFeEDTA) is used. This value was 20.7 mg/kg (15.4–41.2) when WFP6 was excluded from the analysis.

**TABLE 6:** Iron, vitamin A, and iodine content of composite positive fortified samples of wheat flour, oil, and salt samples, respectively, by origin, type of food vehicle, and domestic producers.

Food vehicle, micronutrient	Sample type (*n*)	Micronutrient content^1^	*P* value^2^
Wheat flour, iron^3^ (mg/kg)St. 54–66 mg/kg and 36–44 mg/kg if using NaFeEDTA	Domestic, all (29)	20.6 (16–56.25)	0.0327
	Imported (11)	15.4 (12–20.3)	
	Domestic, mills^4^ (20)	27.4 (19.1–58.5)	0.0008
	Imported (11)	15.4 (12–20.3)	
	Bread flour, all (9)	41.2 (26.1–57.3)	0.0017
	Nonbread flour (33)	16.1 (12.2–21.5)	
	Bread flour, mills^4^ (9)	41.2 (26.1–57.3)	0.0097
	Nonbread flour ( 24)	17.5 (14.4–25.5)	
	WFP1 (12)	57.1 (22.9–61.9)	0.0025
	WFP2 (3)	26.1 (23.6–26.8)	
	WFP3 (2)	21.5 (15.1–28)	
	WFP4 (2)	13.8 (10.4–17.2)	
	WFP5 (1)	41.2 (41.2–41.2)	
	WFP6 (9)	12.2 (11–16.6)	
Oil, vitamin A^5^ (mg RE/kg)St. 11–24 mg/kg	Domestic (9)	14.2 (13.5–17.4)	0.0437
	Imported (44)	9.3 (5.8–12.8)	
	Palm oil (22)	10.4 (8.3–12.3)	0.4171
	Peanut oil (2)	9.7 (3–16.3)	
	Sunflower oil (20)	12.7 (5.4–15.3)	
	Soybean oil (5)	13.5 (7.6–21.1)	
	Unspecified	8.5 (3–10.5)	
	OP1 (3)	14.2 (13.5–14.2)	0.1753
	OP2 (2)	22.2 (21.1–23.3)	
	OP3 (1)	16.3 (16.3–16.3)	
	Others (3)	3 (3–17.4)	
Salt, iodine^6^ (mg/kg)St. 20–60 mg/kg	Domestic, all (34)	38.9 (17.4–61.9)	0.4495
	Imported ( 5)	32.3 (24.9–33.8)	
	Fine salt (9)	31.7 (18.5–55.3)	0.4663
	Coarse salt (33)	48.1 (12.7–61.9)	
	SP1 (3)	48.1 (25.4–69.3)	0.4926
	SP2 (1)	66.6 (66.6–66.6)	
	Others (30)	33.0 (12.7–61.3)	

Abbreviation: NaFeEDTA, sodium ferric EDTA; WFP, wheat flour producer.

1 Values are median (IQR).

2 *P* value of Kruskal–Wallis or Wilcoxon rank-sum’s tests based on valid entries.

3 Milligram of iron per kilogram of wheat flour.

4 Analysis only considers composite samples from mills and excludes composite samples from WFP6, a repackaged flour for baking mixes, supplied by an unidentified mill(s).

5 Milligram retinol equivalent of vitamin A per kilogram of oil.

6 Milligram of iodine per kilogram of salt.

A total of 72 oil composite samples were prepared from qualitatively positive oil samples. For efficiency, 76.4% (*n* = 55) of composite samples encompassing all the oil brands tested positive in the qualitative test (*n* = 50), regardless of type, were tested for vitamin A. The median vitamin A content of oil composite samples was 10.8 (5.8–14.2) mg RE per kg of oil when the Senegalese standard requests 11–24 mg/kg.

For salt, 46 composite samples were prepared from qualitatively positive samples. Five samples were excluded from the analysis, including 4 where we were unable to quantify potassium iodide and 1 incorrectly composited sample. The median iodine content of composite samples was 33.3 (18.5–58.2) mg iodine per kg of salt whereas the Senegalese standard requires 20–60 mg/kg.

Supplemental Figures 1–3 describe the average iron, vitamin A, and iodine content per brand for wheat flour, oil, and salt, respectively, in comparison to the national FS.

### Fortification quality

**FIGURE 1:**
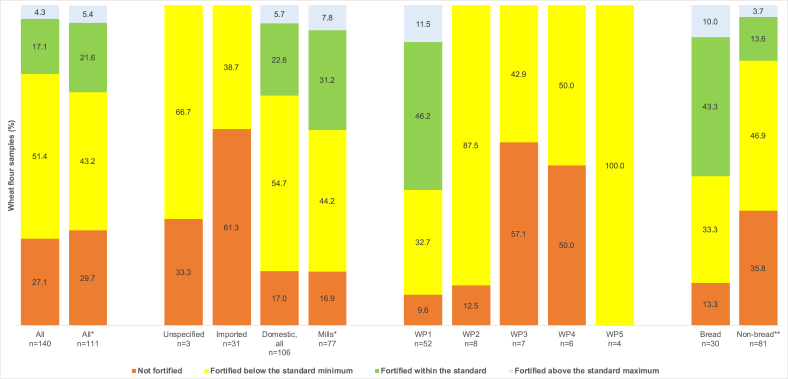
Fortification quality of wheat flour available in the marketplace in relation to the Senegalese national wheat flour fortification standard; ∗analyses exclude samples from WFP6 (*n* = 29); ∗∗all wheat flour other than bread flour. WFP, wheat flour producer.

Figure 1 describes the fortification quality of WF with iron. Overall, 21.6% of WF samples complied with the standard. None of the imported flour was fortified to standard, whereas 39% of WF from domestic mills met standards or with a higher content. Wheat flour was exclusively from WFP1 and represented 57.7% of its samples, which means that only WFP1 was approaching compliance with the standard. The proportion of bread flour that met standards was significantly higher than that of the nonbread flour (*P* <0.001). Only 11.7% (*n* = 3) of the wheat flour brands met the standards, all of them also produced by WFP1.

**FIGURE 2:**
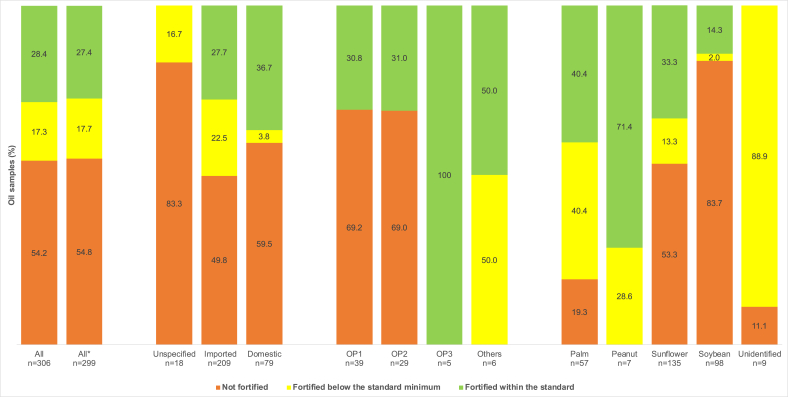
Fortification quality of the oil available in the marketplace in relation to the Senegalese national oil fortification standard; ∗analyses excluding 1 brand with samples originating both locally and abroad. OP, oil producer.

The fortification quality of oil with vitamin A is presented in Figure 2. Twenty-eight percent of the oil samples (28.4%) were fortified within standards. The proportion of oil samples fortified according to the standard is comparable between domestic production and imports. However, the latter has a significantly higher proportion of samples fortified below the minimum of the standard (*P* <0.001). Only oil samples from OP3 were fortified to standards, whereas only one-third of OP1 and OP2 samples were fortified, but to standards. Other national producers had as many fortified oil samples below the standard minimum as within the standard. The proportion of fortified samples within the standards was higher for peanut oil, which is produced exclusively in Senegal, than for other types. In terms of brands, 25 of the 69 (36.2%) found on the market were fortified within the required standards.

**FIGURE 3:**
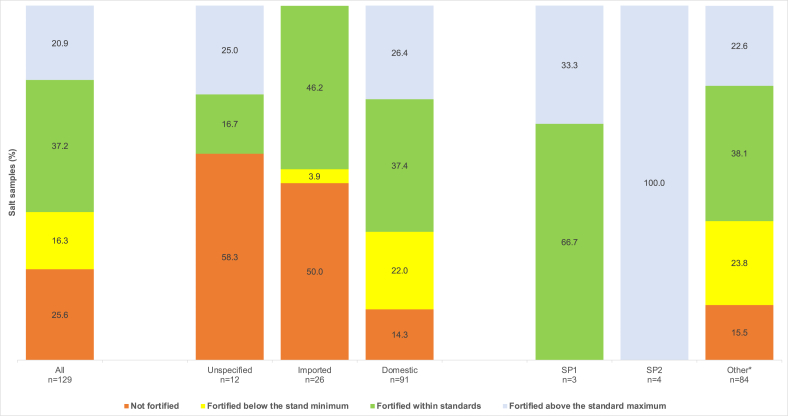
Fortification quality of salt available on the market in relation to the Senegalese national salt fortification standard; ∗other salt producers. SP, salt producer.

Overall, 63.8% of the salt was fortified with iodine within the national standard (37.2%) or with a higher content (Figure 3). The proportion of salt fortified within the standard did not differ according to origin. An analysis of domestic production showed that noniodized salt and salt iodized below the standard minimum were exclusively found among small-scale producers. However, there was a significant proportion of salt fortified above the standard maximum, regardless of the type of domestic producer. Of the 31 salt brands available on the market, 41.9% were fortified according to standards.

## Discussion

This study aims to address an evident information gap regarding the availability and compliance of fortified WF, oil, and salt at the market level in Senegal with respect to mandatory fortification standards. Such information is essential for determining the performance of these programs, and then informing national fortification efforts, targeting food vehicles and companies that should be prioritized for improvement. This could contribute to positive health impacts through increased availability and consumption of quality fortified foods. The findings of the present study indicate an availability of fortified foods with the majority of WF, oil, and salt samples found on the market fortified with iron, vitamin A, and iodine, respectively. However, the iron content of WF and that of vitamin A in oil were below the lower limits of the applicable standards. Only the iodine content of salt was within the recommended range. Consequently, only 17.1%, 28.4%, and 37.2% of the collected WF, oil, and salt samples, respectively, complied with the required FS.

Over 70% of WF and 65.7% of salt samples collected from the marketplace in this study were domestically produced, but only a quarter (24.7%) of the oil. These figures significantly differ from those recently reported in a USAID AFFORD assessment of the fortified food industry in Senegal (USAID AFFORD and Nicolas du Payrat [Altai Consulting], “Assessing Large-Scale Food Fortification Opportunities in Senegal: Industry feasibility of LSFF assessment,” 2023), which reported as domestically produced 99%, 60%, and 99%, respectively, for WF, oil, and salt. This difference may stem from the different objectives and methodologies employed in both studies. The USAID AFFORD industry assessment utilized a desk review and key informant interviews to describe the industry landscape, whereas our study focused on a sample of food vehicles, which, although reasonably representative of their availability in the Senegalese market, does not offer market share data. Our study found samples from all major domestic mills, with the exception of 1 WF producer that holds 5% of the market share in Senegal. The study also revealed a wide variety of brands (27 for WF, 69 for oil, and 31 for salt).

### Wheat flour

The median iron content in WF was 17.5 mg/kg (14.3–26.8, as the IQR), which is significantly lower than the standard requirement of 54–66 mg/kg using ferrous sulfate or fumarate, and 36–44 mg/kg if using NaFeEDTA. In addition, only ∼1 of 4 (26.1%) WF samples from the mills were fortified to standards. These findings indicate that much work remains to improve fortification compliance and the quality of iron fortification in soft WF. The proportion of WF meeting the standards in our study was higher than the 30% reported in a FACT survey from Côte d’Ivoire [53].

Although domestic wheat flour exhibited a significantly higher median iron content and was more likely to approach meeting the FS compared with imports, both showed median concentrations well below the standard minimum. Improving domestic WF compliance is crucial, as it could reduce nutrient inadequacy from 54% to 46% and from 38% to 33% among women of reproductive age and children, respectively [54]. This is particularly important because 99% of the WF market in Senegal is domestically produced. However, only 1 domestic mill met satisfactory compliance, suggesting that local producers face challenges such as sourcing quality premixes, high costs, insufficient internal quality control, or not being equally or consistently monitored for compliance with FS by the responsible government agency. This may be due to a number of factors, including low priority and capacity (insufficient human resources and/or training of existing human resources or a lack of resources required to carry out and succeed in this control) for enforcement [17].

More specifically, our findings also suggest an increase in the share of nonfortified samples (from 4% to 13.3%) and a decrease in the iron content of bread flour from 44 mg/kg to 27.4 mg/kg, compared with the 2014 data [42]. This could be attributed to various factors, including the cessation of partners financial support to millers’ purchase of premixes, as usual in the early stages of the fortification program [55]. However, it is important to note the methodological differences between the current and the past study (including sampling techniques and analytical methods). Specifically, the iron concentration in the earlier study was measured using an iCheck Iron photometer, which tends to yield systematically lower values compared with the AAS, the reference method we used, although both are concordant [56].

Furthermore, the quality of fortification differs according to the type of WF. For instance, almost 43.3% of bread flour is fortified to the standard (assuming that ferrous sulfate or fumarate is being used), compared with only 13.6% for nonbread flour. This discrepancy may be attributed to manufacturers’ continued adherence to the requirements of the previous version of the standard, which only covered bread flour, to the detriment of the ECOWAS standard that has been adopted as the Senegalese standard, which covers all types of WF, and currently in force. This hypothesis is consistent with USAID AFFORD’s policy and industry assessments which found conflicting understanding of the term “*farine de blé tendre*” (soft WF) as mentioned in the standard. Wheat flour millers interpreted the term as referring only to flour used in bread production, whereas policy stakeholders felt that the term included refined flour regardless of its intended use [USAID AFFORD and Nicolas du Payrat (Altai Consulting), “Assessing Large-Scale Food Fortification Opportunities in Senegal: Industry feasibility of LSFF assessment,” 2023; USAID AFFORD and Christophe Guyondet (Freelance Consultant), “Assessing Large-Scale Food Fortification Opportunities in Senegal: Policy enabling environment assessment,” 2023].

### Oil

In the case of oil, fortified samples had an average vitamin A content of 10.8 mg retinol equivalent (RE)/kg (5.8–14.2), which is just around the minimum content specified by the standard (11–24 mg/kg). This value is comparable to a previously reported level in Dakar (10.5 mg RE/kg oil), but higher than the national average of 7.6 mg RE/kg oil reported in the same study [41]. The overall compliance with FS was low (28.4%), which is similar to the 27.5% compliance reported previously. Furthermore, the proportion of nonfortified oil samples nearly doubled from 23.9% in 2014 to 44.6% in this study. These findings suggest that, over the past decade, compliance of oil to vitamin A FS has not improved.

One possible explanation for this could be that the oil industry is either not fortifying or is only fortifying with the minimum required amount of vitamin A. The high cost of premixes and their nontax-exempt status may contribute to this situation [55]. Additionally, vitamin A losses between oil production and distribution, estimated between 53% and 75% [41], could play a significant role in reducing the final levels of fortification. According to the ASN, fortified edible oil should be packaged in opaque, hermetically sealed containers to ensure optimum vitamin A stability. Exposure to sunlight and excessive heat during storage, transport, and distribution can degrade vitamin A. However, our study found that over 95% of oil samples were packaged in transparent bottles and came from open markets, where storage conditions typically expose them to sunlight and heat. Notably, oils packaged in dark plastic bottles were all fortified, in contrast to <50% of those packaged in clear bottles. This suggests that producers who invest in dark plastic bottles are more likely to comply with FS and/or that packaging quality is a critical factor that should be considered in improving the quality of oil fortification.

Furthermore, our results showed that the median vitamin A concentration in imported oil [9.3 mg/kg (5.8–12.8 mg/kg)] was below the standard minimum, whereas locally produced oil had a median of 14.2 mg/kg (13.5–17.4 mg/kg), which is within the standard range. As a result, imported oil had a higher proportion of samples that were fortified below the required minimum. This indicates that oil importers may not be ensuring their products meet Senegal's FS, or that regulatory monitoring at the borders is insufficient. Among the 3 major local producers, only 1 had satisfactory compliance, whereas the other 2 produced oils that were compliant when fortified but had a significant proportion of nonfortified samples (about two-thirds). In contrast, small producers seemed to make a greater effort to fortify their oil, although not enough to ensure full compliance across all samples.

### Salt

The mean content in iodized salt samples was 33.3 mg iodine/kg (18.5–58.2), which reflects a relatively good level of compliance with the standard requirement of 20–60 mg/kg. The median iodine content also falls within the specified range, irrespective of the origin of production. This value is higher than the 2.2 mg/kg reported in Bénin [57]. Additionally, the proportion of salt brands meeting the iodization standard (38.7%) was higher than that observed in Burkina Faso (27%) who is adhering to the same standards [20].

Our results revealed that most formal salt processing facilities iodized their salt production almost entirely, some even adding higher amounts of iodine than necessary. In contrast, small salt processors exhibited significantly lower compliance, with only 38.1% of samples meeting the standards. Inconsistent iodization by artisanal producers may be attributed to a lack of awareness regarding FS, insufficient adherence to proper iodization practices, or challenges related to storage in market conditions. Moreover, the obsolescence of iodization equipment, which entails additional maintenance costs, probably impacts producers’ profitability and could, therefore, have a negative impact on their fortification efforts.

Despite these challenges, the Government of Senegal, with support from partners such as Nutrition International, UNICEF, and the World Food Programme, has made efforts to strengthen the Universal Salt Iodization program. These efforts included providing production units, capacity-building initiatives, and establishing a potassium iodate supply system [58,59]. Nevertheless, the high cost of premixes and associated taxes (similar to WF and oil sectors) remain significant obstacles. In this respect, one of the main recommendations arising from the recent Forum des Affaires dans l’Industrie du Sel au Sénégal was that the cost of potassium iodate should be shared between manufacturers, producers, and public authorities [60]. However, in our view, the solution may be to promote the industrialization of this portion of the salt sector. Poor fortification results from small-scale/artisanal producers highlight the difficulties in implementing fortification outside of large-scale fortification. Improving the fortification of this part of domestic salt, which accounts for 40%–45% of the country's salt market, could potentially reduce iodine inadequacy.

### Fortification labeling

Regarding the presence of a fortification label, 79.6% of WF, 97.9% of oil, and 87.1% of salt were found to have one, indicating that despite the weakness of compliance with fortification requirements, producers are familiar with the labeling requirements. However, the presence of a fortification label does not guarantee compliance with regulations. Our findings revealed that 17.2% of WF, 44.8% of oils, and 15.6% of salts with a fortification label were not fortified.

### Programmatic implications

Our results imply several key actions to ensure effective fortification of soft WF and oil, as well as salt iodization. First, we present those that need to be implemented by the government, followed by those that the private sector should address.

#### Government

The government could initiate a dialog with domestic producers or carry out a value chain analysis of the reasons why they are fortifying or not fortifying, as has been done previously in Bangladesh [61]. In addition, a thorough review of the existing regulatory framework and related processes at production, import, and market levels would be beneficial. A feasible approach to these actions would be to proceed to an overall program impact pathway (PIP) analysis of the country's food fortification program, as implemented in Cameroon [62]. A PIP analysis is an approach that uses the theory of change to identify key inputs, processes, and outputs in a program's causal chain. This will help identify and/or confirm existing gaps and understand barriers to compliance at all levels and uncover comprehensive and sustainable strategies to enhance the fortification quality of fortified food vehicles.

In the meantime, the government should prioritize the recruitment and training of sufficient personnel while ensuring the continuous professional development of existing staff. This should include providing the necessary material and financial resources to facilitate the effective implementation of regulatory monitoring. Such measures would contribute to: *1*) increasing the frequency and improving the quality of audits of quality assurance/quality control processes, as well as inspections of the fortification quality of WF and oil directed at local producers to varying degrees; *2*) enhancing the assessment of the fortification quality of imported oil at border entry points [including a review of certificate of analysis (CoA)], taking into account the primary source of importation, whether at the port of Dakar, land borders, or both; and *3*) ensuring regular and consistent inspections at the market level.

More specifically, adherence to FS for oil should also encompass the verification of appropriate packaging and storage conditions to ensure the preservation of vitamin A at the market level, especially in open market environments. It is also important to confirm the stability of vitamin A in the different types of oil in Senegal.

For salt iodization, targeted support should be provided through access to iodization equipment, regular maintenance of this equipment, and training in proper iodization techniques. Furthermore, incentivizing the industrialization of salt production will be essential for scaling up the effectiveness of iodization efforts.

To improve overall compliance with WF FS, it would be crucial to enhance awareness among domestic producers to mitigate misunderstandings regarding the requirements for WF fortification and to promote the effective application of the FS.

The government should also consider implementing penalties for noncompliance, alongside incentive-based measures such as tax exemptions or subsidies for the acquisition of premixes, particularly for small and medium-sized local producers. These measures would help reduce financial barriers to compliance and encourage broader and more consistent adherence to mandatory fortification regulations. Additionally, recognizing and rewarding exemplary practices could further incentivize compliance with FS.

#### Private sector

Our results also imply the following actions to be implemented by the private sector. Domestic producers should *1*) advocate for tax exemption on premix, *2*) improve the internal quality assurance and control systems of domestic WF and oil fortification with iron and vitamin A, respectively, including procurement of high-quality premixes from trustworthy providers and offering better training for staff, and *3*) especially for oil, comply with the quality standard for packaging. In regard to importers, it is incumbent upon them to ensure that the oil they introduce into the country meets the required standards by being stricter with their suppliers on fortification requirements (e.g. demand a CoA issued by an accredited laboratory).

### Study limitations

Our findings have 2 major limitations. First, our sample did not allow us to obtain a nationally representative situation that accounts for potential differences in the quality of micronutrient fortification of food vehicles by residency (urban compared with rural), as in the 2014 FACT survey, which distinguished, for example, a higher concentration of vitamin A and a lower proportion of nonfortified oil in urban strata compared with rural ones. This may limit the generalization of our results at the national level. Second, we measured total iron in WF and compared it with the iron fortification standard, which refers to added iron. West Africa's food composition table estimates intrinsic iron in refined WF at 20 mg/kg [63], whereas our results were suggestive of intrinsic iron levels ∼10 mg/kg. Considering the standard's very narrow range, the presence of intrinsic iron can have a significant impact on the classification of a sample. What is more, the total iron concentration we found, without deducting intrinsic iron, was already below the lower limit of the standard. Thus, our WF fortification results are actually an underestimate of noncompliance and consequently an overestimate of overall compliance, indicating that the situation is probably worse than described. This points to the need to have standards with a sufficiently wide compliance interval to take into account all types of variation that can occur during the production of a fortified food: mill production, laboratory analysis, and intrinsic values.

In conclusion, this study provides an update on the fortification quality of refined vegetable oil with vitamin A, WF with iron, and salt with iodine in Senegal. It demonstrates that although fortification of these products is already taking place, and the programs count with standards and the awareness of authorities, the private sector, and the public, quality gaps and challenges still need to be addressed. Disparities in the extent of fortification and quality gaps by origin of production, among domestic producers, and sometimes, by type of food vehicle have implications for targeting improvements in fortification quality.

## Author contributions

The authors’ responsibilities were as follows – NID, AD, DP, BLT, PMN, MHF: conceptualized and designed the study; M-MAD: contributed to the design of the study; MHF, M-MAD: conducted the data collection and curation, conducted statistical analysis and interpretation, and wrote the manuscript; MADS: conducted food sample analysis; PMN: contributed to food sample analysis; DP, PMN, BLT: contributed to data analysis and interpretation; NID, DP, BLT, PMN, AD, AB, OMM: critically revised the manuscript; and all authors: read and approved the final manuscript.

## Disclaimer

The views expressed in this article are those of the authors and do not necessarily reflect the views of USAID or the U.S. Government.

## Data availability

The data used and/or analyzed during the current study are available from the corresponding author on reasonable request.

## Funding

This work was supported by the U.S. Agency for International Development (USAID; cooperative agreement number 7200AA22LE00002; USAID Advancing Food Fortification Opportunities to Reinforce Diets).

## Conflict of interest

The authors report no conflicts of interest.
