# Supplementary material for: Assessing the Fortification Quality of Refined Vegetable Oil with Vitamin A, Wheat Flour with Iron, and Salt with Iodine: Findings from a Market Assessment in Senegal, West Africa
Source: Curr Dev Nutr. 2025 Apr 9;9(5):107440. doi: 10.1016/j.cdnut.2025.107440 (PMC12099449; doi:10.1016/j.cdnut.2025.107440)
Supplement: Multimedia component 1 [file mmc1.pdf]

Title: Assessing the fortification quality of refined vegetable oil with vitamin A, wheat flour with iron, and salt with iodine: findings from a market assessment in Senegal, West Africa

Mane Hélène Faye et al.

Supplementary material

Supplementary Table S1: Origin of production of wheat flour, oil and salt brands[1]

Food vehicle	Origin of production	n	%
**Wheat flour[2]**	**Domestic**		
	WFP1[3]	5	41.7
	WFP2	2	16.7
	WFP3	2	16.7
	WFP4	1	8.3
	WFP5	1	8.3
	WFP6[4]	1	8.3
	**Imported**		
	Germany	2	15.4%
	France	8	61.5%
	Germany and France	1	7.7%
	Belgium	1	7.7%
	Turkey	1	7.7%
**Oil[5]**	**Domestic**		
	OP1[6]	4	40.0
	OP2	2	20.0
	OP3	1	10.0
	Other domestic producers	3	30.0
	**Imported**		
	Turkey	16	29.1
	Indonesia	11	20.0
	Ukraine	5	9.1
	Morocco	3	5.5
	Malaysia	3	5.5
	Spain	2	3.6
	United Arab Emirates	1	1.8
	Ghana	1	1.8
	From multiple countries	12	21.8
	Unspecified country	1	1.8
**Salt[7]**	**Domestic**		
	SP1[8]	1	5.25
	SP2	1	5.25
	Other	17	89.5
	**Imported**		
	France	6	85.7
	Spain	1	14.3

[1]Values are n and %

[2]Domestic: 12 brands, Imported: 13 brands

[3]WFP: Wheat flour producer

[4]WFP6 is a repackaged flour for baking mixes, supplied by an unidentified mill(s) and not a wheat flour mill. Only referred to WFP for the purpose of the paper

[5]Domestic: 10 brands, Imported: 55 brands

[6]OP: Oil producer

[7]Domestic: 19 brands, Imported: 7 brands

[8]SP: Salt producer.

Supplementary Table S2: Distribution of wheat flour, oil, and salt brands by types of food vehicle and marketplace categories[1]

	Characteristics	Imported[2] n (%)	Domestic[3] n (%)
**Wheat flour**	**Type of wheat flour**		
	Bread flour	1 (7.7)	6 (50)
	Pastry flour	2 (15.4)	3 (25)
	All-purpose flour	4 (30.8)	-
	Available as multiple types	3 (23.1)	1 (8.3)
	Other types	3 (23.1)	2 (16.7)
	**Marketplace categories[4]**		
	Open markets	2 (15.4)	7 (58.3)
	Hypermarkets	11 (92.3)	6 (50)
	Retail outlets	-	5 (41.7)
	Wholesale outlets	-	2 (16.7)
	Bakeries	-	5 (41.7)
**Oil**	**Type of oil**		
	Palm	17 (30.9)	1 (10.0)
	Peanut	0 (0)	2 (20.0)
	Sunflower	26 (47.3)	2 (20.0)
	Soybean	2 (3.6)	3 (30.0)
	Available as multiple types	6 (10.9)	1 (10.0)
	Unspecified	4 (7.3)	1 (10.0)
	**Marketplace categories[4]**		
	Open markets	51 (92.7)	8 (80.0)
	Hypermarkets	14 (25.5)	6 (60.0)
	Retail outlets	18 (32.7)	3 (30.0)
	Wholesale outlets	6 (10.9)	6 (60.0)
	Bakeries	2 (3.6)	0 (0)
**Salt**	**Type of salt**		
	Fine salt	4 (57.1)	8 (42.1)
	Coarse salt	0 (0)	2 (10.5)
	Available as multiple types	3 (42.9)	9 (47.4)
	**Marketplace categories[4]**		
	Open markets	2 (28.6)	16 (84.2)
	Hypermarkets	7 (100)	4 (21.1)
	Retail outlets	1 (14.3)	6 (31.6)
	Wholesale outlets	1 (14.3)	4 (21.1)
	Bakeries	0 (0)	1 (5.3)

[1]Values are n and %

[2]Imported: 13, 55, and 7 brands for wheat flour, oil, and salt, respectively

[3]Domestic: 12, 10, and 19 brands for wheat flour, oil, and salt, respectively

[4]Frequency of appearance of brands by marketplace categories

Supplementary Table S3: Fortification compliance of wheat flour, oil, and salt brands with iron, vitamin A, and iodine, respectively, by origin of production and type of food vehicle[1]

		Not fortified	Fortified	Inconclusive[2]
**Wheat flour**	**Origin of production**			
	Domestic (n=12)	0 (0)	7 (58.3)	5 (41.7)
	Imported (n=13)	4 (30.8)	4 (30.8)	5 (38.4)
	**Type of wheat flour**			
	Bread (n=7)	0 (0)	6 (85.7)	1 (14.3)
	Other[3] (n=15)	3 (20.0)	4 (26.7)	8 (53.3)
	Available as bread and other type (n=2)	0 (0)	1 (50.0)	1 (50.0)
**Oil**	**Origin of production**			
	Domestic (n=10)	1 (10.0)	4 (40.0)	5 (50.0)
	Imported (n=55)	16 (29.1)	28 (50.9)	11 (20.0)
	**Type of oil**			
	Palm oil (n=19)	2 (10.5)	14 (73.7)	3 (15.8)
	Peanut oil (n=2)	0 (0)	2 (100)	0 (0)
	Sunflower oil (n=28)	12 (42.9)	12 (42.9)	4 (14.3)
	Soybean oil (n=6)	4 (66.7)	0 (0)	2 (33.3)
	Unspecified[4] (n=6)	1 (16.7)	5 (83.3)	0 (0)
	Available as multiple types (n=8)	0 (0)	0 (0)	8 (100)
**Salt**	**Origin of production**			
	Domestic (n=19)	1 (5.3)	14 (73.7)	4 (21.1
	Imported (n=7)	3 (42.8)	2 (28.6)	2 (28.6)
	**Type of wheat flour**			
	Fine (n=16)	4 (25.0)	11 (68.8)	1 (6.2)
	Coarse (n=4)	3 (75.0)	1 (25.0)	0 (0)
	Available as multiple types (n=11)	1 9.1)	4 (36.4)	6 (54.5)

[1]Values are n (%)

[2]Brands with both fortified and non-fortified samples

[3]All wheat flour types other than bread flour. This analysis excludes the brand from WFP6

[4]Unspecified type of oil.

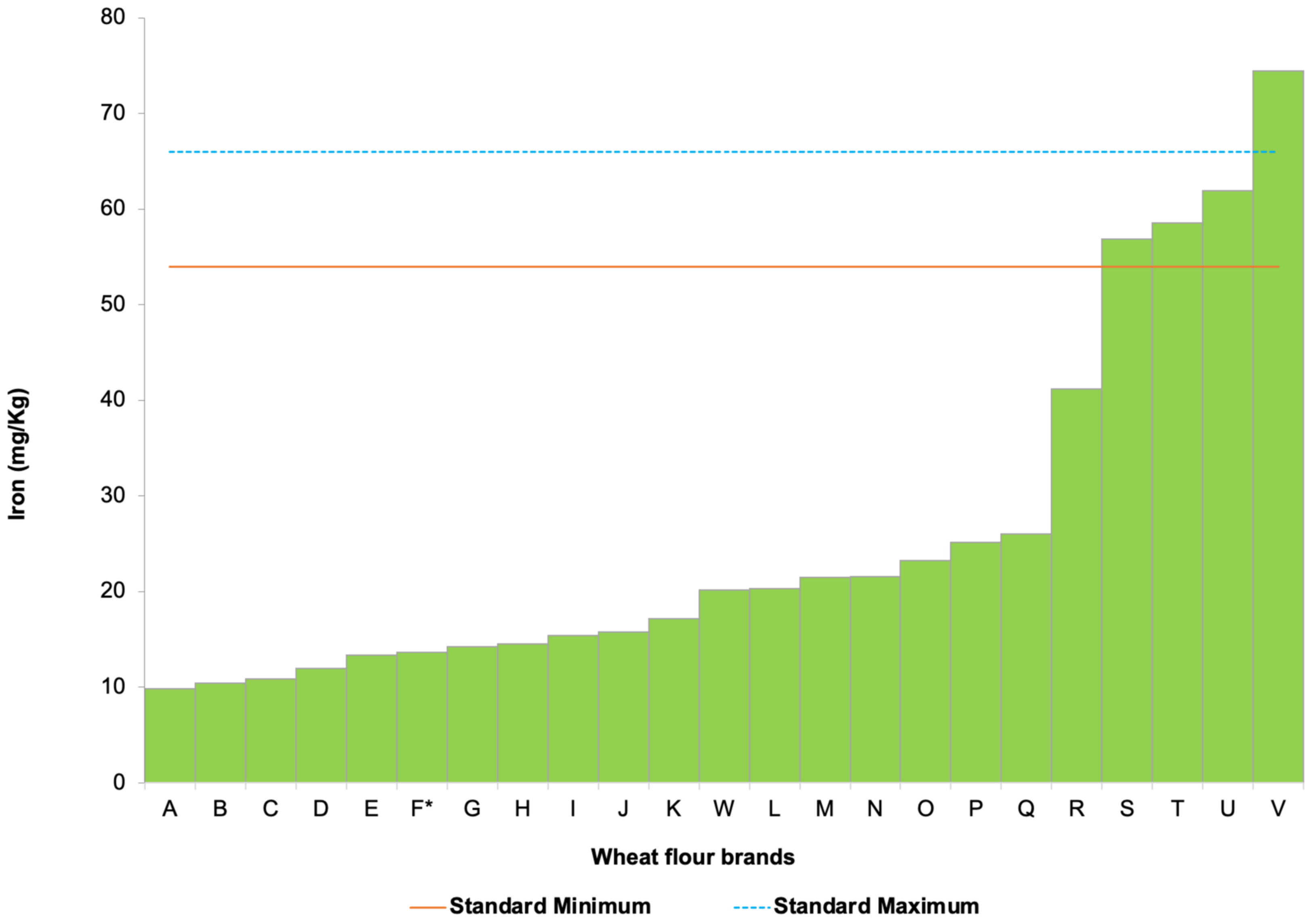

Supplementary Figure S1: Wheat flour. Average iron content of wheat flour brands compared to Senegal iron fortification standards (60 ± 10% mg/Kg); *Brand produced by WFP6.

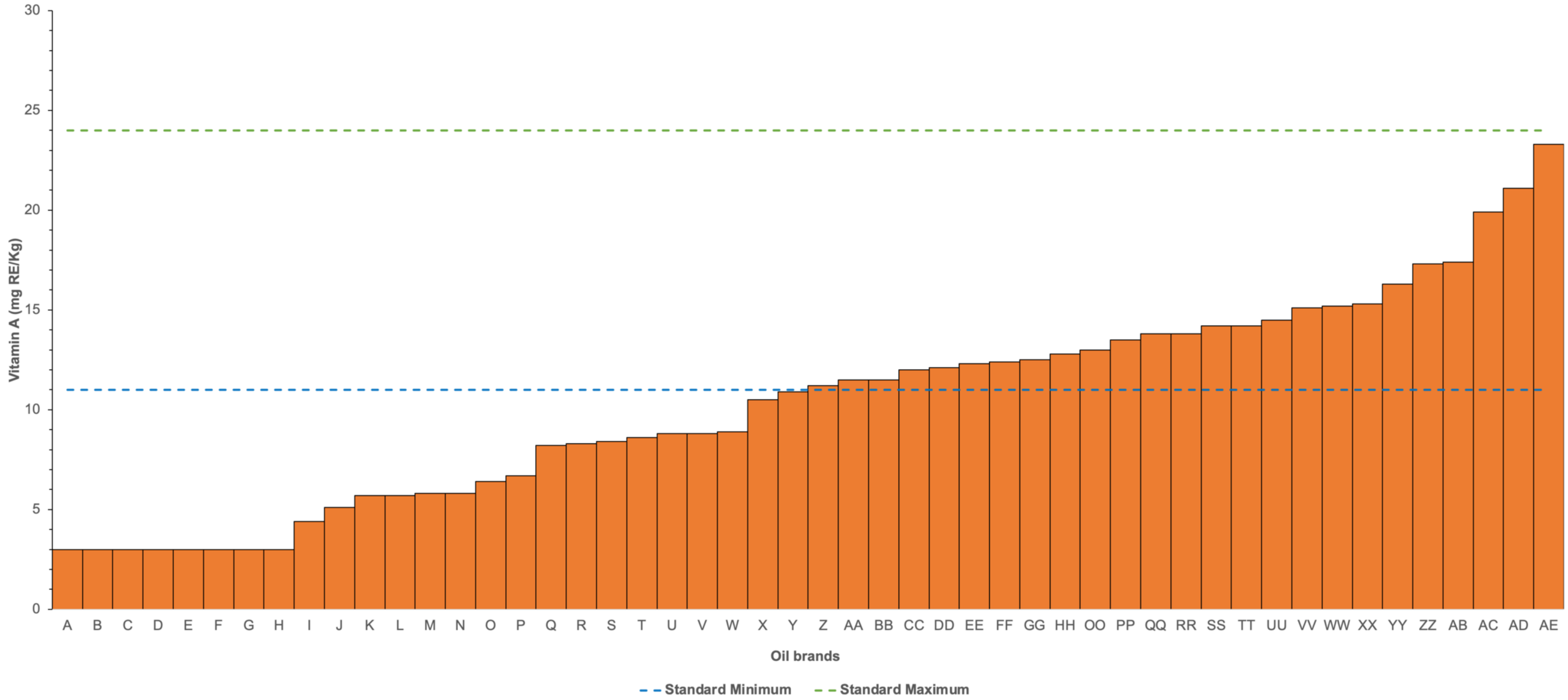

Supplementary Figure S2: Refined edible oil. Vitamin A content per brand in comparison to the national fortification standards (11-24 mg RE/kg).

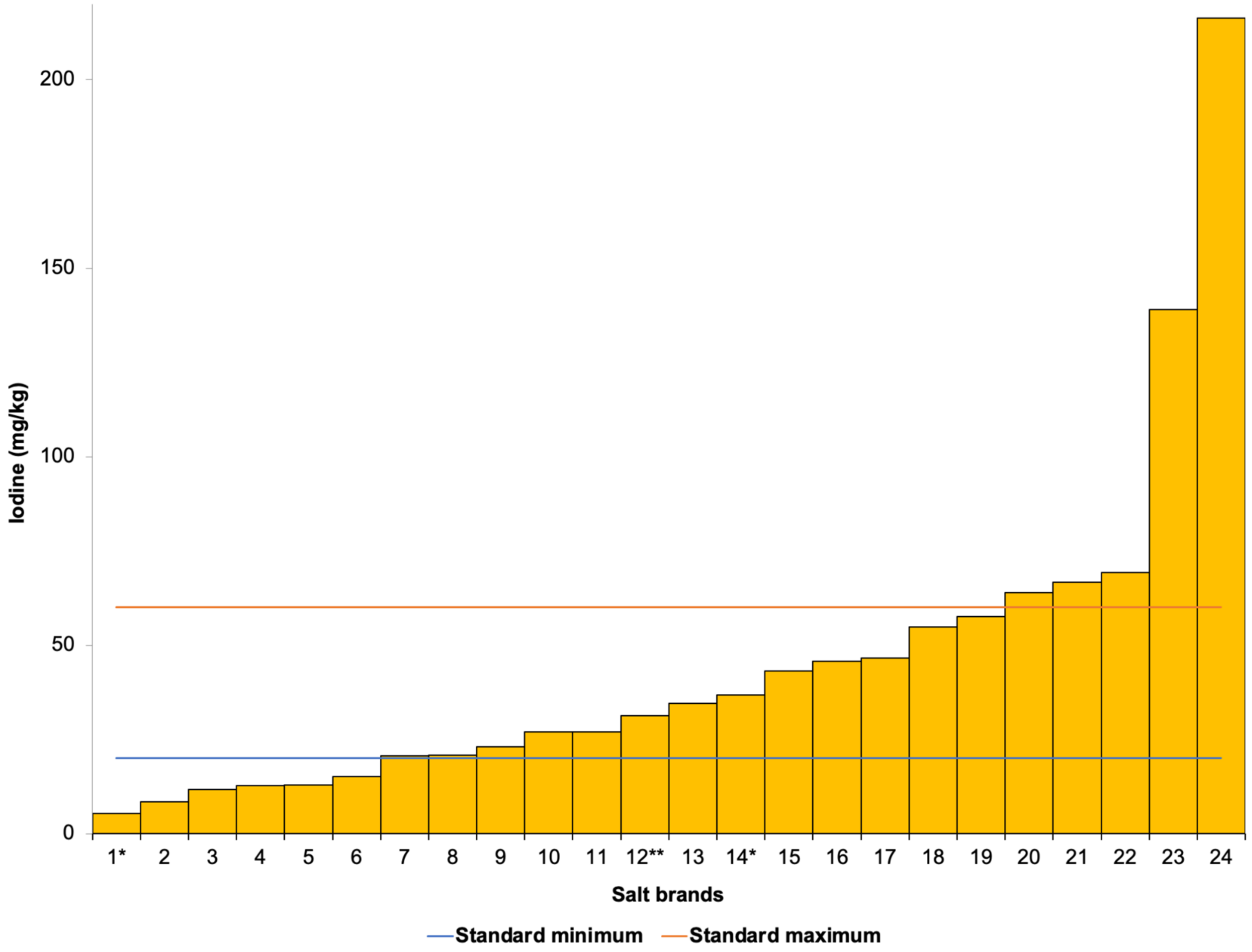

Supplementary Figure S3: Salt. Average iodine content of salt brands compared to Senegal iodine fortification standards (mg/Kg); *Unbranded samples from known producers; **Unbranded sample from unknown producer.
